# Brain Lateralization and Cognitive Capacity

**DOI:** 10.3390/ani11071996

**Published:** 2021-07-03

**Authors:** Lesley J. Rogers

**Affiliations:** School of Science and Technology, University of New England, Armidale, NSW 2351, Australia; lrogers@une.edu.au

**Keywords:** individual lateralization, directional lateralization, cognitive capacity, parallel processing, social behaviour, visual attention, vertebrates, invertebrates, commissures, strength of lateralization

## Abstract

**Simple Summary:**

We used to think of brains as symmetrical, functioning in the same way on the left and right sides, but we now know that this is not so. From the small brains of insects to variously sized brains of vertebrates, including humans, the left and right sides process information differently and control different patterns of behaviour. This is known as lateralization. Lateralized brains can carry out different functions simultaneously on the left and right sides (e.g., monitoring for predators while searching for food). Avoiding duplication in this way increases cognitive capacity. This paper considers the cognitive advantages of two kinds of lateralization. The first, known as individual lateralization, means that most individuals in a species are lateralized, roughly half in one direction and the other half in the other direction. The second type of lateralization, known as directional or population lateralization, means that most individuals have the same direction of lateralization. Directional lateralization is important for social behaviour but, as this paper argues, it may not increase cognitive capacity any more than does individual lateralization. Strength of lateralization is discussed and so is the communication between the left and right sides of the brain.

**Abstract:**

One way to increase cognitive capacity is to avoid duplication of functions on the left and right sides of the brain. There is a convincing body of evidence showing that such asymmetry, or lateralization, occurs in a wide range of both vertebrate and invertebrate species. Each hemisphere of the brain can attend to different types of stimuli or to different aspects of the same stimulus and each hemisphere analyses information using different neural processes. A brain can engage in more than one task at the same time, as in monitoring for predators (right hemisphere) while searching for food (left hemisphere). Increased cognitive capacity is achieved if individuals are lateralized in one direction or the other. The advantages and disadvantages of individual lateralization are discussed. This paper argues that directional, or population-level, lateralization, which occurs when most individuals in a species have the same direction of lateralization, provides no additional increase in cognitive capacity compared to individual lateralization although directional lateralization is advantageous in social interactions. Strength of lateralization is considered, including the disadvantage of being very strongly lateralized. The role of brain commissures is also discussed with consideration of cognitive capacity.

## 1. Introduction

The left and right sides of the brain are specialised to attend to different information, to process sensory inputs in different ways and to control different types of motor behaviour. This is referred to as hemispheric specialization or simply as brain lateralization. Such division of function between the hemispheres has long been known in humans and considered to increase ‘neural space’ [1] or cognitive capacity [2]. Additionally, in humans, there are clear structural differences between the hemispheres (summarised in [3]). Using functional magnetic resonance imaging of human brains, Gotts et al. [2] provided evidence that the left hemisphere has stronger interaction within itself (intrahemispheric), whereas processing by the right hemisphere involves greater involvement of both hemispheres (interhemispheric) [2].

Lateralization has now been well-documented across species, ranging from the small brains of insects [4] to early vertebrates, birds and mammals [5,6,7]. This ubiquity of brain lateralization suggests that, regardless of the absolute size of the brain, its capacity and efficiency may be increased by not duplicating all functions on the left and right sides. By avoiding duplication of information processing on the left and right sides of the brain, more “cognitive space” is available to carry out different types of processing. In other words, lateralization of brain function should be able to increase cognitive capacity without the more costly process of increasing brain size.

From studies on a variety of species and using a range of techniques, a general pattern of lateralization has been constructed. As conceptualized by Andrew [8], the left hemisphere controls sustained response to targets, whereas the right hemisphere is specialized for response to potent releasers of innate responses. The left hemisphere directs attention to specific categories of stimuli, often learned categories, and controls feeding responses [9,10,11,12,13]. The right hemisphere has broad attention to a wider variety of stimuli and especially to novel stimuli [14] and to predators (shown in toads [15]; a lizard [16]; a marsupial [17]; and in dogs [18,19]. The right hemisphere is also specialized for expressing intense emotion [18,20,21], for handling geometric information [22] and for dealing with social interactions [23,24,25]. In line with the latter, and as shown in chicks, attack and copulatory behaviour are functions of the right hemisphere and they can be elicited readily once inhibition of the right hemisphere by the left hemisphere is suppressed or removed [9,26]. Similar right hemisphere activation of attack has been shown also in toads [27,28], frogs [12], lizards [29], penguins [30,31], Australian magpies [32], horses [33] and gelada baboons [34]. It appears, therefore, that these lateralized expressions of behaviour are common to a wide range of vertebrate species.

The first evidence of brain lateralization in non-human species was discovered in avian species; by inhibiting protein synthesis in the left or right forebrain hemisphere of chicks (*Gallus gallus domesticus)* at critical stages of development and then investigating the longer-term effects on behaviour [35], summarised in [36], and by lesioning specific regions of the left or right hemisphere of songbirds and assessing the effects on song production [37]. Then, in rats, it was shown by assessing the effects on behaviour of ablation of the left or right hemisphere [38].

Later, lateralization was revealed simply by testing animals monocularly [39] and this has become a standard method to investigate lateralized behaviour. In species with eyes positioned on the sides of their head, and thus with little overlap of the visual fields, visual information goes mainly to the contralateral side of the brain (discussed further in Section 7). Hence, lateralized differences can be revealed by applying an eye-patch and comparing the performance elicited when the left versus the right eye is seeing [39,40]. The eye-system of the open (seeing) eye, comprising the visual inputs mainly to the contralateral hemisphere, has predominant control of behaviour. Laterality revealed by monocular testing is evident even in small sample sizes, indicating that it must have a significant role in biologically relevant situations.

Within a species, the strength of lateralized responses can vary. Some individuals exhibit strong laterality, while others have weaker laterality or no significant laterality. However, weak or absent laterality expressed in motor behaviour (e.g., hand or limb preference) may not mean that the brain is itself less lateralized for cognitive processing. It is rather a matter of whether one hemisphere alone controls a particular behaviour, as is the case in strong laterality, or that the other hemisphere is involved to some degree.

What does this mean in terms of cognitive capacity? Even when both hemispheres participate in the control of behaviour and interhemispheric control occurs, cognitive capacity is increased as long as each hemisphere performs different computations of available information. Provided each hemisphere is processing information differently and leading to different outcomes, cognitive capacity is increased. By contrast, if both hemispheres are processing information in the same way, and hence duplication is occurring, there is no enhancement of cognitive capacity. In short, more lateralization means greater cognitive capacity.

## 2. Advantage of Having a Lateralized Brain

Since lateralization is widespread across animal species, it follows that there must be situations in which having a lateralized brain confers an advantage and enhances survival. Logically, this should apply to situations in which the animal has to use both hemispheres in parallel to carry out different functions.

This was shown first in domestic chicks by presenting a silhouette of a predator approaching the chick from its left or right side while the chick was pecking, with focused attention, at a patch of grain and mealworms [41]. Two groups of chicks were tested with both eyes seeing: one group with lateralized visual behaviour, ensured by exposing the eggs to light before hatching [9,42], and the other group lacking visual lateralization, achieved by incubating the eggs in the dark [43]. As discussed above, light-exposed chicks are lateralized for use of the left hemisphere (right eye) in searching for food and for use of the right hemisphere (left eye) to respond to predators. Latency to detect the predator was scored as the time between presentation of the ‘predator’ and the time when the chick stopped pecking, giving a startle call and, usually, twitching its head. In lateralized chicks, the latency was shorter when the predator’s image approached on the chick’s left side than when it did so on the right side. The latency of non-lateralized chicks was the same on both sides and not different from the latency of the lateralized chicks on their poorer, right side. The longer time taken by the non-lateralized chicks to detect the predator was not due to reduced levels of fear. In fact, after catching sight of the predator, the non-lateralized chicks were more disturbed by its presence than were the lateralized chicks, as shown by the fact that they produced more distress calls, and continued to do so even after the predator was no longer present [41].

To investigate this difference between lateralized and non-lateralized chicks further, they were tested on a dual task requiring search for grains against a distracting background of pebbles, and at the same time they were presented with a silhouette of a predator moving overhead. Chicks with lateralization of visual function performed both aspects of this task better than chicks lacking lateralization [44]. The lateralized chicks learnt to find grain scattered amongst pebbles, whereas the non-lateralized ones were unable to do so, and the lateralized chicks detected the predator sooner than the non-lateralized chicks. Once they had detected the predator, the non-lateralized chicks were more disturbed by it, as shown by distress calling and being less able to ignore it in order to continue pecking for food [20,44]. Similar results were found also when the lateralized and non-lateralized chicks were tested in groups [45]. Clearly, the lateralized chicks had the capacity to detect the predator while feeding and then to monitor it as they continued to feed. They achieved this increased cognitive capacity by using the different processing abilities of each hemisphere simultaneously; the left hemisphere to discriminate grains from pebbles and the right hemisphere to detect and respond to the predator.

Similar results have been found in both fish and a primate species tested on dual tasks. Topminnow fish, *Girardinus falcatus*, had to feed on shrimps in the presence of an on-looking predatory fish. Topminnow fish with stronger lateralization, assessed by turning bias in a runway, were faster at catching the shrimps than were the fish with no lateralization [46]. Similarly, lateralized female topminnows are able to find food efficiently while avoiding a male attempting to mate with them, whereas non-lateralized females are less able to do so [47].

In marmosets, *Callithrix jacchus,* strength of hand preference for simple reaching was used as an indication of the degree of lateralized *use* of the hemispheres (i.e., not the pattern of brain lateralization *per se* but its expression in motor behaviour). The marmosets were tested on a dual task in which a model predator was introduced to the testing room when the marmoset was performing a discrimination search-task for a favourite food, mealworms [48]. For two types of model predator, a stuffed bird moved overhead or a snake-like model moved on the floor below the marmoset, there was a significant, negative correlation between strength of hand preference and latency to detect the predator. Marmosets with stronger hand preferences detected the presence of the model predator sooner than did marmosets with weaker hand preferences [48]. No difference in latency to detect the predator was found when the marmosets had to perform only one aspect of the task: viz., detection of the predator when they were not feeding at the same time. Hence, the relationship between strength of hand preference and latency to detect the predator emerged only when increased cognitive capacity was needed in the dual task.

A cognitive advantage of being lateralized has even been shown in the invertebrate, larval antlion, *Myrmeleon bore* [49]. Compared to antlions without side-biases, antlions that have significant side-biases in the righting response have enhanced ability to learn to associate a vibrational cue with disappearance of prey. Although motor laterality may not be an accurate measure of brain lateralization, as I have discussed previously for vertebrate species, it is also worth noting a study showing that desert locusts (*Schistocerca gregaria*) with stronger lateralization of forelimb use to reach across a gap perform fewer errors of reaching than do locusts with weaker lateralization [50].

These examples provide evidence that lateralization of the brain increases cognitive capacity, in the sense that it increases the brain’s ability to handle more information at any given time. Apparently, this works only for temporarily paired stimuli demanding simultaneous use of the separate specialisations of the hemispheres. If both stimuli needed to be processed within the same hemisphere, interference may occur and cognitive capacity would be reduced. Indeed, when tested in the dual task of pecking at grain while a predator was moved overhead (discussed previously), the behaviour of chicks without visual lateralization indicated that they became increasingly confused, or disturbed, by the dual task since their ability to find food grains scattered among the pebbles deteriorated as the task continued [44]. It appears that the chicks’ ability to function well in the dual task was compromised by an inability to separate the required functions into different hemispheres, as Gotts et al. [2] found in a study of humans.

Since animals in the natural environment must be vigilant for predators at the same time as they are feeding, these findings indicate enhanced survival of lateralized animals. A study of larval coral reef fish, *Acanthurus triostegus*, supports this; when exposed to a predatory fish, survival was highest in those larvae that used their left eye to monitor the predator compared to larvae with no eye preference or right eye preference [51].

There is more evidence showing that lateralization influences performance in ways that might be advantageous depending on the context. Cichlid fish, *Geophagus brasiliensis*, for example, are said to be bolder if they are lateralized, as indicated by shorter latency to emerge into an unfamiliar environment [52], and lateralized fish show shorter latency to escape when stimulated by dropping a cylinder into their holding tank [53]. Hence, lateralized fish are not only more exploratory than are non-lateralized fish but they are also faster to respond to danger. Another study on a fish, the convict cichlid, *Archocentrus nigrofasciatus,* showed an association between aggression and strength of laterality: aggression was higher in more strongly lateralized males but the reverse was so in females [54].

Other evidence shows the advantage of having a lateralized brain. Pigeons with stronger laterality, measured as strength of eye preference, learn to find grains among pebbles better than non-lateralized pigeons and there is a linear association between these two factors [55]. Sailfish with stronger laterality for attacking prey are more successful in prey capture [56]. Budgerigars with stronger side bias in preening display enhanced discrimination performance [57]. The latter result has some similarity to that found in cats: testing cats on novel tasks requiring them to open a lid and reach inside to obtain a reward, Isparta et al. [58] found that those using a single preferred paw were better at solving the problems.

Degree of laterality is also associated with behaviour in tasks other than those directly testing cognitive ability. For example, in several tests, including tonic immobility and time to emerge from a box, lateralized chicks are found to be less fearful than non-lateralized chicks [59] and dogs exposed to the sounds of a thunderstorm are less reactive if they have a stronger paw-preference [60]. Across species of parrots, those species with stronger foot preferences for holding food are better able to discriminate seed from pebbles and they also perform better in a string-pulling task [61]. Note that this measure of performance, as a score comparing species, differs from the other studies discussed so far, which have compared variation in laterality within a species. Furthermore, parrot species with stronger foot preferences have larger brains [62], which may be the reason for their better cognitive performance rather than having a lateralized brain per se, although both factors could contribute to better cognitive ability.

Strength of lateralization also affects other cognitive abilities. Lateralized fish, for example, have better numerical abilities than non-lateralized fish [63,64]. As another example, lateralized chicks tested on the dual task mentioned above retained a memory of discrimination between pebbles and grain on the next day, whereas the non-lateralized chicks retained no memory of the task [44]. However, the inability of non-lateralized chicks to remember may have resulted from inability to attend to the pebble-grain task in the presence of the predator (on the previous day of testing) rather than being a direct association between lateralization and memory formation.

In summary, all of these studies show the advantage of being lateralized in such a way that one hemisphere can take charge of performing a particular task while, at the same time, the other hemisphere takes charge of performing another task. This is evidence that lateralization increases cognitive capacity.

## 3. Tasks Performed Better When Lateralization Is Weak or Absent

Since not all individuals within a species have the same strength of brain lateralization, there may be some contexts in which being less lateralized is an advantage. In contrast to the study on cichlid fish, which reported greater boldness in lateralized fish (see above), Brown and Bilbost [65] found, in rainbow fish, *Melanotaenia nigrans,* that non-lateralized fish are bolder than lateralized fish. As the researchers suggest, boldness is influenced by past experience with predators and may be modulated by fear. Consistent with this, reactivity to stress co-varies with laterality. For example, lateralized sharks react more strongly to stress than do non-lateralized sharks [66] and the same applies to the reaction of lambs to stress [67]. In fact, from research on humans, it seems likely that stress alters interhemispheric integration [68], thereby altering strength of lateralization and cognitive capacity.

These findings indicate that the increased cognitive capacity gained by having a lateralized brain may be associated with heightened stress responses, depending on context. In fact, in the dual task on which lateralized chicks performed better than non-lateralized chicks, it was the non-lateralized ones that were more distressed. Whether this distress translates to higher levels of physiological stress has yet to be determined.

As already mentioned, attack behaviour is a function of the right hemisphere [9]. Nevertheless, as shown in deer, non-lateralized individuals are more likely to engage in successful fights with conspecifics [69]. Additionally, testing damselfish, *Pomacentrus amboinesis*, Chivers et al. [70] found that lateralized individuals were less likely than non-lateralized ones to attack conspecifics when competing for shelter, even though they showed stronger responses to a predator. This result led the researchers to consider that there are costs and benefits of being lateralized. When an animal must attend equally to both sides, they suggest, it would be a disadvantage to be lateralized. In fact, as Dadda et al. [71] showed, in topminnow fish, non-lateralized individuals have an advantage over lateralized individuals in tasks requiring attention to both sides of their body, and hence, requiring the same use of both hemispheres.

## 4. Balance between Being Lateralized or Not Lateralized

Depending on the type of task and its cognitive demands, performance may be better in individuals with no lateralization or, conversely, better in individuals with lateralization. Overall, however, across and within species, lateralization is more common than non-lateralization. Nevertheless, very strongly lateralized individuals may be at a disadvantage. As an example, pheasants with strong foot-preference have a lower rate of survival than pheasants with a weaker strength of foot-preference [72]. In humans, stronger lateralization provides advantages in some but not all tasks [73]. This illustrates the important point that lateralization is largely specific for each different function. While it may be advantageous for some functions, it may confer no advantage or even a disadvantage for other functions.

As Corballis [74] suggests, there may be a trade-off between symmetry and asymmetry of function, but where the balance point lies depends on the behaviour considered (for research on this issue in humans see [75]), the species, sex, stress levels and possibly other factors, as well as genetic. Within a population the strength of bias is maintained as an evolutionary stable strategy [6,21].

This raises a different question: where does the balance between lateralization and non-lateralization lie within any group of animals? Using game theory analysis of a predator–prey model, Ghirlanda and Vallortigara [76] arrived at the conclusion that most but not all individuals in a group or population are lateralized (see also [77] for a similar result using the analysis of a competition-coordination model). As predicted by game theory, and found in studies of animal populations, the proportion of lateralized individuals in a species ranges from 65 to 90% and such biases in populations are stable, meaning the natural selection restores the proportion of left versus right biased individuals whenever there are slight deviations from the species-typical equilibrium point [78]. Although there are examples of published data in which the group bias is greater than 90% (e.g., footedness in some species of cockatoo [61,62]), the sample size tested needs to be considered.

## 5. Population Versus Individual Lateralization

The increased cognitive capacity of brains that carry out different computational or neural processes on each side could be achieved regardless of the direction of the laterality. Despite this, most examples of lateralization discussed so far in this paper are directional, meaning that the direction of the laterality is the same in the majority of individuals in the group or species. In other words, lateralization is not only present at the individual level but also at the level of the population.

There may be ontogenetic reasons for this situation. For example, in the final stages of incubation before hatching, the chick embryo is oriented within the egg so that its right eye is next to the shell and the left eye is occluded by the chick’s body. This posture determines the direction of structural differences in developing visual pathways as a consequence of light stimulation of the right eye only [79]. Hence, light exposure at this critical stage of development leads to a population bias for asymmetry of visual behaviour [9,42].

Whatever the reason for individuals having the same direction of asymmetry, because it is widespread across species, the advantage that it confers must over-ride any potential disadvantages. Population lateralization to detect and respond more readily to predators on the left seems to be disadvantageous since predators are just as likely to approach on the right or the left, unless the predators themselves have population-level lateralization that predisposes them to approach prey from behind and capture them on the predator’s right side. There are examples of such right-side bias in predatory response: the cane toad, for example, strikes at prey once the prey has moved into the toad’s right visual field, whereas prey items are ignored when they are in the toad’s left visual field [11]. A similar result has been found in the music frog [80]. Such preferential use of the right eye in feeding, or predation, originally shown in chicks [35], has also been reported to occur in humpback whales [81] and blue whales [82]. A right-side preference for prey capture by wild stilts has also been reported [83]. Other predators, however, may attack prey to their left or right (individual bias but no population bias), as found to be the case in sailfish [56]. Interestingly, although individual sailfish showed more success in prey capture on their preferred side and those with stronger laterality were more successful, the population showed no side-difference in success of capturing prey, which also implies that the prey were not better at escaping when attacked from their left side, although this has been found in some tests of amphibians [15], a species of marsupial [17] and another species of fish [84].

If population-level lateralization, also called directional lateralization, does have certain disadvantages, these must be less important than the advantages which it bestows. From the evidence discussed so far, it seems that the advantage of population-level lateralization must have something to do with social behaviour because it is in social interactions that it is manifested, as discussed next.

## 6. Social Cognition

The first evidence that directional lateralization is associated with social behaviour came from the study of social hierarchies in groups of chicks with lateralized brains for visual function compared to groups of chicks without this lateralization [85]. Quite rapidly, groups of chicks with population-level lateralization established stable social hierarchies, measured by scoring access to a limited food source, whereas those without laterality failed to form stable hierarchies. In fish also, being lateralized at the population level is associated with social group formation and maintenance. Bisazza et al. [86] tested 16 species of fish, some known to form shoals and others not so. They measured turning behaviour of each fish individually and determined whether each species had individual lateralization or directional lateralization. All of the species that displayed shoaling behaviour were lateralized at the population level, whereas this occurred in less than half of the species that did not shoal [86]. Clearly, a shoal is maintained if individual fish turn together in the same direction. The directional lateralization is essential for this particular aspect of social behaviour.

With particular relevance to lateralized chicks forming stable social hierarchies, young chicks have a quality called transitive inference, a cognitive ability that permits a chick to infer the social rank of another chick by observing it rather than interacting directly with it [87]. Using transitive inference, the animal is able to predict its position in the social group and thereby avoid fights and, as found by Daisley et al. [88], they can make such inferences when they use their left eye but not when they use their right eye. These examples demonstrate that the right hemisphere is specialised for functions essential to social behaviour.

The ability recognise familiar from unfamiliar conspecifics is a function of the left eye and right hemisphere, as shown in chicks [23,89] and fish [90]. Chicks have been shown to respond consistently to social signals when using their left eye [87,88] and, provided they are using their left eye, they can learn to avoid pecking a distasteful bead by observation of the behaviour of a conspecific [91]. These are aspects of social cognition and they involve specialisations of the right hemisphere.

Specialisation of the right hemisphere to assess and respond in social situations is seen also in mammals. Sheep can recognise faces of other sheep using the right but not the left hemisphere [92] and they also respond to the emotional expression of those faces using the right hemisphere [93]. Considered from the other perspective, the right hemisphere has a dominant role in producing expressions of fear, as shown in rhesus monkeys [94] and marmosets [95]. Moreover, in a wide range of mammalian species, including bats, walruses, whales, dolphins, horses, kangaroos, sheep, deer and bison, maternal animals position their offspring on their left side [96,97,98], meaning that the maternal animal uses her right hemisphere to monitor the offspring. A similar left-side/right-hemisphere preference has been shown in gorillas and chimpanzees for monitoring conspecifics [99]. These examples show that directional bias is a widespread characteristic of social behaviour.

One way to investigate the association between directional lateralization and social behaviour is to compare laterality in social and non-social species. Comparison of different lateralities in several species of bee has provided some information on this topic. The hypothesis tested was that social species should show directional laterality, whereas asocial species should not, although they may show individual laterality. This line of research began with the discovery of directional lateralization in the honeybee, *Apis mellifera*, a highly social species [100]. It has long been known that honeybees can be conditioned to associate a specific odour (e.g., lemon or vanilla) with a food reward (sugar solution). They detect the odour with receptors on their antennae and respond by extending the proboscis. Letzkus et al. [100] discovered that honeybees can learn this task when they use their right antenna but not when they are forced to use their left antenna. Not surprisingly, they can recall memory of the task when the odour is presented to the right, but not the left, antenna, at least, in the short-term, up to an hour after learning [101]. When tested for recall of the memory 6 h or more after learning, they can do so using their left but not their right antenna [101]. Hence, short-term and long-term memory is laid down in different sites, and likely on different sides, of the brain and accessed by the right or left antenna, respectively. These are directional lateralities of olfactory learning and memory recall.

The population bias for olfactory learning is also present in three species of Australian stingless bees [102], all of which are social species, but not in the largely asocial mason bee, *Osmia sp.* [103]. However, what about asymmetry in mason bees when they do happen to interact socially? Discovery of directional lateralization of agonistic responses in honeybees [104] suggested a way to test this since mason bees do interact with each other when they fight. In aggressive encounters, which are particularly intense between females, mason bees do show directional lateralization: both males and females interact more aggressively when they use the left antenna but not when they use the right antenna [105]. Hence, aggressive interaction, which is an aspect of social behaviour, is directionally lateralized. This does not imply anything about a role of lateralization in an individual’s likelihood of success in aggressive interactions, examples of which are discussed below.

This finding supports the hypothesis that population or directional lateralization evolves, or develops, in social interactions and it is, of course, evident in a wider range of behaviour in social species than it is in species that are largely solitary. Even social species may show individual but not population lateralization in behaviour that does not involve social interaction. As an example, Ong et al. [106] found that this is so for direction choice when honeybees are flying through holes in a barrier and, since the bees were tested without other bees being nearby, they were not interacting socially while they made a choice. Individual bees were found to have lateral preferences but about half preferred the right side and the other half preferred the left side. Overall, therefore, population-level asymmetry is present only in social behaviour.

During evolution, social interaction could have selected for alignment of laterality in most individuals. Could this lead to any increase in cognitive capacity? It has long been hypothesised that social interaction contributes to the evolution of increased brain size and cognitive capacity in primates [107] and this hypothesis is supported by evidence that neocortical size correlates with social group size in primates [108] and also in insectivores and carnivores [109]. It is not obvious, however, that aligning the direction of laterality (i.e., directional lateralization) in social animals provides any further increase in cognitive capacity than already gained by having individual lateralization.

In fact, it seems that individual-level lateralization almost certainly evolved first and there was selection for it because it enhanced cognitive ability. Then, as sociality evolved, directional lateralization did so along with it, not because it further enhanced cognitive ability but because it conferred an advantage in social interactions.

Returning to the original research addressing this question, we can see one reason for aligning laterality within a population: in groups of chicks, it pays to be lateralized at the population level because it stabilises the social hierarchy, as discussed above [85]. It also maintains the coherency of a shoal of fish, as also mentioned above [86], and as reported recently, more strongly lateralized fish (measured as eye preference) are more likely to be in the safest, less exposed position in the shoal [110]. In many mammalian species left-side preference for positioning of the young next to the mother, presumably, ensures optimum interaction between mother and young [96,97]. These are social advantages but not necessarily cognitive advantages. In other words, there can be social advantages for aligning direction of laterality [78], without evidence of any cognitive advantage.

In some species directional laterality increases success in aggressive interactions (e.g., in flies [111]) and in other species it decreases the likelihood of success (in deer [69]). As another example of the latter, Schnell et al. [112] found that the majority of giant Australian cuttlefish have a preference to use their left eye during escalated fighting but the minority with a right-eye preference have more success in the outcomes of these fights. However, in mating, most male cuttlefish use their left eye as they approach the female on her right side and it is these males that achieve higher mating success [112], which illustrates that there must be a selective balance between advantage in one behaviour and disadvantage in another. In sage-grouse also, males lateralized with a left-eye preference in aggressive encounters are more successful in mating [113].

Again, these are examples of social advantage of population lateralization but not of superior cognitive capacity. Nevertheless, success in aggressive interactions depends on tactical decisions, and the same may apply to success in mating, and this could reflect higher cognitive capacity. Whether or not this is so must depend on the degree of sensory processing needed to make these decisions and we do not yet know this.

## 7. Interaction between the Hemispheres

So far, I have discussed lateralization revealed by testing animals monocularly and followed the general interpretation, common to most papers in this area of research, that behaviour performed when using the right eye reflects specialisations of the left hemisphere and vice versa. Although this is largely correct, the situation is a little more complex than that.

In species with small binocular fields, visual inputs from one eye are, indeed, processed primarily in the contralateral hemisphere. However, this is does not mean the contralateral hemisphere is used exclusively, although that is often assumed to be the case because it is the parsimonious way to interpret results from monocular tests. This interpretation ignores the fact that some visual inputs go to the ipsilateral hemisphere too, even though these inputs are much less than the visual inputs to the contralateral hemisphere. Additionally, brains have interhemispheric commissures and this is the case in almost all vertebrate species, even though these commissures are considerably smaller than the major interhemispheric commissure, the corpus callosum, in humans [114,115].

The avian brain, for example, has an interhemispheric commissure, the anterior commissure, and two commissures in the thalamus, the tectal and posterior commissures. In addition, each eye sends at least some inputs that recross the midline (decussate) to the ipsilateral hemisphere. Although we tend to interpret the behavioural differences expressed when the left eye or the right eye is used as reflecting the specialisation of the right or left hemisphere, respectively, in each case the other hemisphere is not without some input and, potentially, has some role in processing sensory information, albeit less than the hemisphere contralateral to the seeing eye. It follows, therefore, that lateralization not only enhances the brain’s cognitive capacity by allocating different processing of information to each hemisphere but also by utilising at the same time, and to some degree, the differing cognitive abilities of each hemisphere. This means that, even though one hemisphere may take a dominant role in processing and controlling response, the other hemisphere is not “silent” and may provide some important contrasting analysis of inputs.

Recent research in Xiao and Güntürkün [116] has studied the role of the anterior commissure in the pigeon tested on a colour-discrimination task. This commissure connects the arcopallial regions of the left and right hemispheres. The researchers blocked the activity of the arcopallium of one hemisphere temporarily and recorded the activity patterns of the neurons in the acrcopallium of the other hemisphere while the bird performed the colour discrimination task. A clear asymmetric effect was found: blocking the activity of the left arcopallium had a greater effect on recordings obtained from the right acropallial neurones than vice versa. Hence, interhemispheric transfer via the anterior commissure is greater from the left to the right than from the right to the left. As Xiao and Güntürkün [116] argue, the neurones in this commissure are largely excitatory and, hence, the left side should step up activity of the right side, thereby acting as a balance against the dominant role of the left hemisphere in colour discrimination. To understand this more completely, it is necessary to know that, in pigeons, the left hemisphere receives visual inputs from both eyes (the tectofugal visual system), whereas the right hemisphere receives inputs mostly from the left eye [117,118]. In domestic chicks, it is the other visual system (the thalamofugal system) that is lateralized: in this visual system, the Wulst region of the right hemisphere receives strong inputs from both eyes, whereas the left Wulst receives input mainly from the right eye [79]. A recent study [119] has shown lateralized integration of visual information in the Wulst regions of the chick brain.

Returning to the study on pigeons [116], although the left hemisphere of the pigeon has a dominant role in colour discrimination, the anterior commissure may provide some tempering of this asymmetry before the animal makes a response. Further research is needed to confirm this interpretation.

The tectal commissure, connecting the optic tectum on one side to its counterpart on the other side, and the posterior commissure may have similar tempering roles on the input of visual information, at least in the chick. When the tectal and posterior commissures of the chick are sectioned, asymmetry of behaviour emerges [120]. Presented with a small red bead, chicks with the commissures sectioned pecked much more when they were using their right eye than when they were using their left eye. In fact, when using their right eye, the chicks pecked more and more each time the red bead was presented to them. No such asymmetry was seen in sham operated chicks or unoperated controls. Hence, without the commissures the brain functions asymmetrically and with the commissures this asymmetry is not seen, presumably because the left hemisphere suppresses the right via the tectal or posterior commissure. That release from control and expression of asymmetry occurs also for attack and copulation behaviour in chicks. Both attack and copulation responses are controlled by the right hemisphere and released from inhibition by the left hemisphere following treatment of the left hemisphere with cycloheximide [9] or when the right, but not the left, eye is occluded [121].

These studies suggest that, although the hemispheres have different ways of processing information and, indeed, each handles a different package of information, the brain can make some adjustments for these differences via the commissures. These adjustments may go some of the way in explaining why individuals vary in the strength of lateralization. It could be these commissures which set the balance discussed above in Section 4.

We know that this balance changes during early development [122] and it is altered by stress [66,67,68,123,124]. More research will, hopefully, elucidate the mechanism(s) by which one hemisphere can assume dominant control of a particular behaviour (strong lateralization) or, as Reddon and Hurd [125] explain it, there is a “consensus of action of the two hemispheres” (weak lateralization). Reddon and Hurd [125] suggest an alternative route by which one hemisphere may override the other and so assume dominance, or fail to do so, as the case may be, and that route involves the habenula nuclei of the epithalamus.

The point relevant to discussion of cognitive capacity is that different sensory inputs to each side of the brain, followed by different types of neural processing are mechanisms of achieving increased cognitive capacity, and interhemispheric and other commissures play a role in balancing or coordinating these left-right differences.

## 8. Conclusions

Cognitive capacity is increased when each hemisphere can be used independently, at the same time. Examples of this ability in different species have been discussed. Other research on rhesus macaques has led to the conclusion that the two hemispheres have independent capacities, which are limited by competition for sensory encoding rather than by a failure of memory formation or recall [126].

Strength of lateralization varies between individuals, as does cognitive capacity. This variation must depend on the task considered and a range of factors play a role, including developmental processes and the selective advantage/disadvantage of having a lateralized brain.

There is a need to investigate a broad range of lateralities within individuals to see what functions are associated in terms of lateralization and what functions are lateralized independently. I realise that this is being studied in humans [127,128,129] and there are some studies of it in non-human species, discussed in this paper, but more examples are needed. Furthermore, cognitive capacity related to laterality in sensory modalities other than vision needs to be investigated, as well as potential interaction between laterality in the different sensory modalities.

Whereas being lateralized confers cognitive advantage to individuals, aligning the direction of lateralization in the majority of individuals of a population, or species, seems unlikely to enhance cognitive capacity to any greater degree than does individual lateralization. Hence, selection for directional (population) lateralization is dependent on social interactions, and not enhancement of cognitive processing. While social behaviour might increase cognitive capacity of species via evolution of increased brain size, occurring with larger group size, as hypothesised by Dunbar [107,108,109], or by the possibility of group-consensus decisions [130], it does not do so by brain lateralization *per se*.

## Data Availability

No need.
